# Sleep quality assessment in intensive care: actigraphy vs. Richards-Campbell sleep questionnaire

**DOI:** 10.5935/1984-0063.20190145

**Published:** 2020

**Authors:** Hana Locihová, Karel Axmann, Katarína Žiaková, Dagmar Šerková, Simona Černochová

**Affiliations:** 1 Department of Nursing, Jesseniuss Faculty of Medicine in Martin, Comenius University in Bratislava, Slovakia.; 2 AGEL Educational and Research Institute (VAVIA), Prostějov, Czech Republic.; 3 Department of Anaesthesiology and Resuscitation and Intensive Care Medicine, University Hospital Olomouc.; 4 Faculty of Medicine and Dentistry, Palacky University Olomouc, Czech Republic.; 5 Department of Nursing and Midwifery, Ostrava, University of Ostrava, Faculty of Medicine, Czech Republic.; 6 Interdisciplinary Intensive Care Unit, Hospital Nový Jičín, Czech Republic.

**Keywords:** Sleep, Actigraphy, Richards-Campbell Sleep Questionnaire, Intensive Care Unit

## Abstract

**Introduction:**

It has been repeatedly shown that sleep of intensive care unit (ICU) patients is fragmented and its architecture is impaired. As sleep disorders have numerous negative effects on the organism, there have been efforts to implement sleep-promoting strategies into practice. When comparing the effectiveness of such measures, sleep quality assessment itself is a considerable problem.

**Objective:**

The study aimed to assess the quality and quantity of night sleep in ICU patients simultaneously with actigraphy (ACT) and the Richards-Campbell Sleep Questionnaire (RCSQ). The secondary goals were to test the performance and effectiveness of the above methods and to verify correlations between selected RCSQ items and actigraph parameters.

**Methods:**

A single-center prospective observational study (20 patients staying in a Interdisciplinary Intensive Care Unit). The quality of sleep was assessed using a Czech version of the RCSQ and ACT. The obtained data were analyzed and their dependence or correlations were veriﬁed by selected statistical tests.

**Results:**

The mean RCSQ score was 47.6 (SD 24.4). The worst results were found for sleep latency (44.4; SD 31.2); the best results were for sleep quality (50.2; SD 29.4). The mean sleep effciency measured with ACT reached 86.6% (SD 9.2); the mean number of awakenings per night was 17.1 (SD 8.5). The RCSQ total parameter with a cutoff of 50 (RCSQ total = 50 good sleep / RCSQ total < 50 poor sleep) was shown to be suitable for discrimination of subjectively perceived sleep quality in ICU patients. However, the study failed to show statistically signiﬁcant relations between subjectively perceived sleep quality (RCSQ) and ACT measurements.

**Conclusion:**

The RCSQ appears to be a suitable instrument for assessing night sleep quality in ICU patients. On the other hand, the study showed a very low level of agreement between subjective sleep quality assessment and objective ACT measurements. The main drawback of ACT is low reliability of obtained data. Further research is needed to determine its role in sleep quality assessment in the ICU setting.

## INTRODUCTION

Sleep disorders are rather common in intensive care unit (ICU) patients^1,2,3^. Studies have shown changes to sleep architecture characterized by increased fragmentation, frequent arousals and more shallow sleep^3,4^. The presence of abnormal sleep patterns in critically ill patients is associated with higher mortality and affects the patient’s clinical outcome^5,6^. Studies have also demonstrated longer daytime sleep, circadian rhythm changes^7,8,9,10^ and a higher incidence of in hospital delirium^11,12^.

A sleep disorder is perceived by patients as an important stressor^13,14^. It has been shown that the condition may persist even after discharge from an ICU and may have adverse neuropsychological effects in survivors discharged from ICUs, manifested as post-ICU syndrome^15,16^. The gold standard for assessing sleep quality in the ICU setting is polysomnography (PSG)^17,18^. The main limitation is that the method is technically demanding, costly and time-consuming. It has been suggested that given the presence of abnormal electrical brain activity (EEG) in critically ill patients, standard American Academy of Sleep Medicine scoring may not be reliable^1,19,20^. Efforts have been made to find other feasible ways of measuring sleep in critically ill patients, with actigraphy (ACT) being a generally recognized and very widespread non-invasive method. Numerous authors^21,22^ claim that the method is undoubtedly beneficial, as evidenced by an increase in the ratio of papers (ACT to PSG) from 1:10 in 1991 to 1:4 in 2009. However, the role of ACT in ICU sleep assessment has not yet been fully established.

This simple method is based on monitoring of motor activity in sleeping patients. Numbers of motions are accelerometrically recorded at preset intervals (15, 30 or 60 seconds). Data are stored in the actigraph memory and analyzed after monitoring is completed. The records may be assessed visually or automatically with software using various algorithms. Choosing an appropriate algorithm is crucial for sensitivity and specificity of the method. The selected algorithm should be calibrated for a particular population of subjects/patients^21,23^. The main benefits of ACT are its technical simplicity and cost-effectiveness. A major limitation, however, is reliability of obtained data. The method is not used on its own; it is often a supportive instrument (e.g. in subjective assessment of the effect of clinical interventions). Subjective assessment of sleep quality in ICUs is mostly performed using questionnaires.

One of the most commonly used instruments is the Richards-Campbell Sleep Questionnaire (RCSQ), containing five items to measure particular sleep characteristics (sleep depth, sleep latency, awakenings, returning to sleep and sleep quality) plus noise, an optional item that is separately evaluated. Each item is scored by using a 0-100 visual analogue scale. The total score is calculated as the mean of all items, with 0 and 100 representing the worst and best sleep, respectively^24^. The psychometric properties of the RCSQ were published earlier. Cronbach’s α was 0.89-0.92 for reliability^24,25,26^ and 0.84 for content validity^27^. The criterion validity (RCSQ vs. PSG) showed a correlation of r=0.58 (*p*<0.001)^24^.

## OBJECTIVE

The primary goal of the study was to assess night sleep quality and quantity in ICU patients using ACT and the RCSQ simultaneously. The secondary goal was to verify the diagnostic properties of the tests and correlations between selected RCSQ items and actigraph parameters.

## MATERIAL AND METHODS

Design

A single-center prospective observational study.

Sample

The sample comprised 20 patients staying in a Interdisciplinary Intensive Care Unit of the Nový Jičín Hospital (12 beds, unselected admission of patients). The inclusion criteria were as follows: full consciousness (Glasgow Coma Scale score 15; orientation to place, time and person), an ICU stay of more than 24 hours, age over 18 years and voluntary consent to participate in the research). The exclusion criteria were previous sleep disorder treatment, neurocognitive dysfunction (cognitive deficit/dementia, organic brain dysfunction), structural brain damage (trauma/stroke), ICU readmission for worsening of the condition, delirium of various etiologies or withdrawal syndrome, administration of sedatives in the previous 24 hours and patient refusal to participate.

### Measurements and data collection

Sleep quality and quantity were simultaneously evaluated using both the RCSQ and ACT. Patient enrollment, actigraph measurements and questionnaire data collection were performed by two trained nurses. The study was conducted from September to December 2018.

### The RCSQ for sleep quality assessment

The questionnaires were filled in once during the hospital stay, on the morning of the second day (between 7a.m. and 9a.m.) after previous nighttime sleep monitoring with ACT. On average, the questionnaires took 2-5 minutes to complete. Patients with visual or other impairments preventing them from completing the questionnaire were helped by the trained nurse.

### Actigraph

The actigraph wGT3X-BT (ActiGraph, USA) was placed on the dominant wrist. The epoch length was set at 60 seconds. The obtained data were processed using software (ActiLife 6.13.3, ActiGraph) with using a specific Cole-Kripke algorithm^28^. Due to the primary focus (night sleep) and pilot design of the study, the monitoring was carried out from 9p.m. to 5a.m. The analyzed actigraph parameters are shown in Table 1.

**Table 1:** Definition of various actigraphy parameters.

Actigraphy parameter	Definition
Time in bed (TIB) [min]	The time between the start and the end of the recording.
Total sleep time (TST) [min]	The total number of minutes scored as "asleep".
Sleep efficiency (SE) [%]	Number of sleep minutes divided by the total number of minutes the subject was in bed multiplied by 100.
Wake after sleep onset (WASO) [min]	The total number of minutes the subject was awake after sleep onset occurred.
Number of awakenings [-]	Number of awakenings per night.
Average awakening length [min]	The average length, in minutes, of all awakening episodes.
Sleep fragmentation index (SFI) [-]	Expressed as a percentage and calculated as the sum of the proportion of all epochs from sleep onset to sleep offset that were mobile.*

* Actigraphy software programs used and was calculated per nighttime (21:00 to 05.00).

### Noise measurement

Additionally, a single 12 hour noise measurement with a sound level meter was performed during a night shift (6p.m. to 6a.m.). Nurses working the night shift were not informed about the noise measurement to eliminate bias the Hawthorne effect, as described by Wickström and Bendix^29^.

### Sleep disorder definition

For the study’s purposes, total RCSQ score of ≥ 50 was used to define good sleep (sensitivity 88.24%, specificity 86.67%) (receiver operating characteristic [ROC] area - 0.92, 95% confidence interval [CI]). Patients with total RCSQ score < 50 were considered to have poor sleep^30^. The cutoff was set arbitrarily, in accordance with literature data mentioned above showing good statistical results.

### Ethical aspects

The study complied with the Declaration of Helsinki and was approved by the Nový Jičín Hospital ethics committee. Respondents participated voluntarily and their anonymity was ensured. Permission to translate the questionnaire into Czech was obtained directly from its author, Prof. K. C. Richards.

### Data analysis

Descriptive statistics (absolute/relative frequency, mean and standard deviation [SD]) were used to analyze demographic and clinical data and to evaluate individual questionnaire items.

Statistical analysis of results was performed using software STATA version 12.0 (STATA Corporation, College Station Road Houston, Texas, USA). The diagnostic power of tests (binary discriminatory ability) was determined as the AUC by Youden’s analysis at a level of significance *p*<0.05, when AUC 0.75-0.92 suggesting good ability, AUC 0.92-0.97 very good and AUC 0.97-1.00 high ability^31^. Relations between selected RCSQ items and ACT were assessed by Spearman’s correlation; Fisher’s exact test was used to examine the dependence of sleep quality on gender. Differences in sleep quality determined by the questionnaire were compared with those measured with an actigraph using non-parametric Mann-Whitney U test (statistical significance *p*<0.05).

## RESULTS

The inclusion criteria were met by 20 patients. The sample comprised nine males (45%) and 11 females (55%). The mean age was 65.7 years (range 18-79 years; SD 14.5), height 166 cm (SD 10.1), weight 80.4 kg (16.7) and body mass index (BMI) 29.2 (SD 6.0). The mean RCSQ total was 47.6 (SD 24.4). Sleep was found to be good (RCSQ≥50) in nine patients (45%) and poor (RCSQ<50) in 11 patients (55%). Among the items, the worst results were found for sleep latency (44.4; SD 31.2); the best results were for sleep quality (50.2; SD 29.4).

The subjectively assessed nighttime noise level was 52.3 (SD 25.0). Single night measurement with a sound level meter, the noise level was determined to be 44.9 DbA (SD 7.2). The highest (53.6 DbA) and lowest (34.1 DbA) noise levels were recorded at 5a.m. and 1a.m., respectively.

ACT yielded the following results: in all cases, the time in bed was standard, namely 480 minutes (8 hours, from 9p.m. to 5a.m.). The total sleep time (TST) was 415.6 minutes (SD 43.9). Sleep efficiency reached 86.6% (SD 9.2), with good sleep quality being defined as efficiency higher than 85%. The mean number of awakenings per night was 17.1 (SD 8.5), with an average length of 3.6 min (SD 1.3). The mean wake after sleep onset (WASO) was 64.5 minutes (SD 43.9). The sleep fragmentation index (SFI) was 40.4 (SD 15.8) (Table 2). The discriminatory ability of individual RCSQ items to identify subjectively assessed good quality sleep was studied and expressed as the AUC. The RCSQ total parameter (mean of 5 items) confirmed high quality discriminatory ability (AUC 1.00; SD 0; *p*<0.001). Returning to sleep showed highest discriminatory ability of items (AUC 0.995; SD 0.0071; *p*<0.001). Another three items were found to be good or very good: awakenings (AUC 0.929; SD 0.0634; *p*<0.001), sleep quality (AUC 0.914; SD 0.0637; *p*<0.001) and sleep latency (AUC 0.909; SD 0.0818; *p*<0.001). For only two items, the AUC was below 0.75; those were sleep depth (AUC 0.707; SD 0.137; *p*=0.065) and noise (AUC 0.551; SD 0.139; *p*=0.358).

**Table 2:** Descriptive statistics of major characteristics and variables of the sample (n=20).

	Variable	Mean ± SD
**Patient**	Age [years]	65.7 ± 14.5
	Height [cm]	166.0 ± 10.1
	Weight [kg]	80.4 ± 16.7
	Body mass index[kg.m^-2^]	29.2 ± 6.0
**RCSQ**	Sleep depth [mm]	48.6 ± 24.0
	Sleep latency [mm]	44.4 ± 31.2
	Awakenings [mm]	47.1 ± 26.5
	Returning to sleep [mm]	47.8 ± 27.2
	Sleep quality [mm]	50.2 ± 29.4
	RCSQ total [mm]	47.6 ± 24.4
	Noise [mm]	52.3 ± 25.0
**Actigraphy**	Sleep efficiency [%]	86.6. ± 9.2
	TST [min]	415.6 ± 43.9
	WASO [min]	64.5 ± 43.9
	Number of awakenings [-]	17.1 ± 8.5
	Average awakening length [min]	3.6 ± 1.3
	SFI [-]	40.4 ± 15.8
**Noise level [DbA]**		44.9 ± 7.2

RCSQ: Richards-Campbell Sleep Questionnaire; TST: Total sleep time; WASO: Wake after sleep onset; SFI: Sleep fragmentation index.

In a similar manner (AUC), the ability to identify good quality sleep was expressed for individual ACT parameters. None of the studied parameters reached satisfactory discriminatory ability (AUC>0.75). For individual ACT parameters, the following values were recorded (in a descending order): the average awakening length (AUC 0.707; SD 0.137; *p*=0.065); SFI (AUC 0.576; SD 0.147; *p*=0.303); sleep efficiency (AUC 0.535; SD 0.14; *p*=0.4); TST (AUC 0.535; SD 0.14; *p*=0.4); WASO (AUC 0.535; SD 0.14; *p*=0.4) and number of awakenings (AUC 0.515; SD 0.142; *p*=0.458) (Table 3).

**Table 3:** Receiver operating characteristics of Richards-Campbell Sleep Questionnaire and actigraphy parameters for discrimination of patients with poor (n=11) and good sleep (n=9).

	Variable	Cutoff value	Youden's statistic	AUC (SD)	p-value	Sensitivity [%]	Specificit y [%]	Accura cy [%]
**RCSQ**	Sleep depth	≤ 3.59	0.576	0.707 (0.137)	0.065	90.9	66.7	80
	Sleep latency	≤ 30.00	0.818	0.909 (0.0818)	< 0.001	81.8	100	90
	Awakenings	≤ 50.00	0.778	0.929 (0.0634)	< 0.001	100	77.8	90
	Returning to sleep	≤ 45.00	0.909	0.995 (0.0071)	< 0.001	90.9	100	95
	Quality of sleep	≤ 49.00	0.707	0.914 (0.0637)	< 0.001	81.8	88.9	85
	RCSQ total	≤ 45.60	1	1(0)	< 0.001	100	100	100
	Noise	≤ 35.00	0.232	0.551 (0.139)	0.358	45.5	77.8	60
**ACT**	Sleep efficiency	≥ 90.00	0.212	0.535 (0.14)	0.4	54.6	66.7	60
	TST	≥ 432.00	0.212	0.535 (0.14)	0.4	54.6	66.7	60
	WASO	≤ 48.00	0.212	0.535 (0.14)	0.4	54.6	66.7	60
	No. awakenings	≥ 30.00	0.273	0.515 (0.142)	0.458	27.3	100	60
	Av. awakening length	≤ 3.59	0.576	0.707 (0.137)	0.065	90.9	66.7	80
	SFI	≤ 43.73	0.374	0.576 (0.147)	0.303	81.8	55.6	70

ACT: Actigraphy; RCSQ: Richards-Campbell Sleep Questionnaire; AUC: Area under curve; TST: Total sleep time; SD: Standard deviation of the mean; WASO: Wake after sleep onset; SFI: Sleep fragmentation index; No. awakenings: Number of awakenings; Av. awakening length: Average awakening length.

Also studied was the dependence of sleep quality (RCSQ total ≥ 50) on demographic parameters. Sleep quality was subjectively perceived as lower by patients with high body weight (83.5±14.1kg vs. 66.6±20.4kg; *p*=0.005), tall individuals (172±7.45cm vs. 159±8.98cm; *p*=0.005) and those with high BMI (33.4±8.49kg/m2 vs. 26.1±3.53kg/m2; *p* **<**0.001). Age was not found to be a statistically significant factor (69.±8.29 vs. 61.3± 20; *p*=0.59) (Table 4). There was a statistically significant correlation (*p*<0.01) between gender and sleep quality. In ICUs, males had significantly poorer sleep than females (Table 5).

**Table 4:** Actigraphy parameters with poor (n=11) and good sleep (n= 9).

Variables	Mean ± SD		Difference
	*Poor sleep (n=11)*	*Good sleep (n=9)*	*p-value^a^*
Age [years]	69.2 ± 8.29	61.3 ± 20	0.59
Height [cm]	172 ± 7.45	159 ± 8.98	0.005
Weight [kg]	83.5 ± 14.1	66.6 ± 20.4	0.005
Body mass index (BMI) [kg.m-2]	33.4 ± 8.49	26.1 ± 3.53	< 0.001
Sleep efficiency [%]	86.4 ± 10.34	86.8 ± 8.69	0.82
Total sleep time (TST) [min]	415 ± 49.6	416 ± 41.7	0.82
Wake after sleep onset (WASO) [min]	65.2 ± 49.6	63.6 ± 41.7	0.82
Number of awakenings [-]	19.1 ± 9.83	14.7 ± 7.04	0.94
Average awakening length [min]	3.22 ± 0.876	3.97 ± 1.64	0.13
Sleep fragmentation index (SFI) [-]	41.7 ± 17	38.9 ± 16.1	0.59

a The differences between groups were evaluated using a robust Mann-Whitney U test.

**Table 5:** Gender differences between poor (n=11) and good sleep (n=9).

Gender	n (%)		Difference
	*Poor sleep (n=11)*	*Good sleep (n=9)*	*p-valuea*
Male	8 (88.9)	1 (11.1)	
Female	3 (27.3)	8 (72.7)	<0.01

^a^ The differences between groups were evaluated using Fisher's exact test.

When assessing differences in individual ACT parameters between groups with different subjective sleep quality (poor vs. good), no statistically significant difference was found: sleep efficiency (86.4±10.34 vs. 86.8±8.69%; *p*=0.82); TST (415±49.6 vs. 416±41.7 minutes; *p*=0.82); WASO (65.2±49.6 vs. 63.6±41.7 minutes; *p*=0.82); awakenings (19.1±9.83 vs. 14.7±7.04; *p*=0.94); average awakening length (3.22±0.876 vs. 3.97±1.64 minutes; *p*=0.13) and SFI (41.7±17 vs. 38.9±16.1; *p*=0.59) (Table 4).

In pairs of variables selected from the RCSQ and ACT parameters, dependence was studied using Spearman’s correlation. No pair of the selected variables showed statistically significant dependence: returning to sleep vs. WASO (r=0.0716; *p*=0.7549); awakenings vs. awakenings (r=0.1097; *p*=0.6324) and sleep quality vs. SFI (r=0.0452; *p*=0.8439).

The results suggest a low level of agreement between subjective sleep quality assessment using a questionnaire and objective ACT measurements.

## DISCUSSION

An increasing awareness of the importance of sleep for patients staying in ICUs has prompted the development of various strategies to increase its quality. In many respects, it is rather difficult to satisfy the need for sleep, mainly because its quality is difficult to assess. A major pitfall is selecting a suitable assessment tool. Outcomes of the present study suggest that subjectively perceived sleep quality in ICUs is low. The total RCSQ score was 47.6, meaning poor sleep; this is consistent with results of similar studies^24,25,26^ reporting total RCSQ scores ranging between 47 to 60.

The present study confirmed the benefit of the RCSQ (and its selected items) as a high-quality diagnostic test able to detect good sleep. At the same time, it confirmed that the cutoff defining good quality sleep is total RCSQ score ≥ 50. However, subjective assessment of sleep quality with a questionnaire is difficult to use in ICU patients due to the presence of limiting factors (frequent functional and structural CNS changes, nearly universal administration of sedatives, a high incidence of delirium, etc.) in a considerable proportion (or even the majority) of patients^32,33^. Naturally, there is an effort to seek adequate techniques for objective measurements that may be applicable in ICUs.

An objective alternative to subjective questionnaire methods is to use ACT for sleep assessment. Given its technical simplicity (e.g. compared with PSG that may be considered as the gold standard), the approach is theoretically easy to use in the ICU setting as well. At the same time, however, the benefit and convenience of its use in ICUs is questionable, mainly because of considerably reduced patients’ own voluntary motor activity. This is conditioned, among other things, by their physical condition and further decreased by the effects of CNS-suppressing drugs (opioids, sedation, etc.).

In the present study, ACT results (sleep efficiency 86.6±9.2% and number of awakenings 17.1±8.5) were not fully consistent with literature data. Three other studies with different outcomes were identified: an Australian study^34^ reporting sleep efficiency 73.5±18.5% / number of awakenings 14±8.3, an Indian study^30^ showing sleep efficiency 80.6±7.8% / number of awakenings 5.0±3.1 and a Canadian study^35^ with sleep efficiency as low as 61.3±41.4%, and by far the highest number of awakenings 48.5±34.0. The varied results may be attributed to both the length of ACT recordings (ranging from 7 hours in the Australian study to 8-12 hours in the Canadian study and to 72 hours in the Indian study) and the sample sizes and characteristics.

Given the aforementioned issue of ACT validity in the ICU setting, the study also aimed to investigate the relations between subjective (RCSQ) and objective (ACT) sleep quality assessment. The obtained results, with none of the ACT parameters proving to be a diagnostic test of sufficient quality to identify good sleep (arbitrarily set based on literature data, see above), confirmed the questionable benefit of ACT as a valid diagnostic tool in the ICU setting. By contrast, subjective assessment with the RCSQ (and most of its items) was found to be a high quality diagnostic test (AUC≥0.9)^31^. The aim of the study was to evaluate each single ACT parameters’ ability to detect good sleep. Thus, no detailed conclusions could be made about the validity of ACT as a diagnostic tool for objective sleep quality assessment in ICU in general, because of the lack of one single integrative parameter available analogous to the *RCSQ total* for subjective testing.

Poor sleep quality in patients with higher body weight (and BMI) is generally consistent with recent findings about the relationship between obesity and sleep quality^36,37^. Additionally, hypotheses about the impact of gender on sleep quality have been proposed and the relationship has been studied. Despite original theoretical assumptions (females sleep badly), the study showed significantly worse sleep quality in males. Possible differences between the genders may be explained by discrepancy in subjective assessment between males and females.

Parameters obtained with ACT showed no statistically significant differences between groups with varied sleep quality, confirming low correlation between subjectively perceived sleep quality and efforts to assess it objectively using ACT. An Indian study^30^ found statistically significant differences between groups for the following items: sleep efficiency (*p*=0.002), WASO (*p*=0.001) and number of awakenings (*p*<0.001). The inconsistency between the studies may be attributed to the design: a bigger sample size (32 vs. 20 participants), different sample characteristics and longer ACT records (72 vs. 8 hours).

The secondary goal was to assess the level of agreement between selected RCSQ items and ACT parameters. However, the study failed to show significant correlations between the selected variables. Although no study comparing RCSQ items with ACT was found, there are studies comparing ACT with other objective techniques (PSG, EEG) in critically ill patients. Once again, they showed that ACT outcomes were overstated and the approach had low sensitivity compared to the above methods. Beecroft et al.^35^ found < 65% agreement with PSG. Van der Kooi et al.^38^ reported 94% sensitivity and only 19% specificity for ACT as compared with PSG. A British study^39^ investigated the relationship between EEG and ACT, showing a low correlation (-0.201).

The weakness of ACT is analysis of obtained data, an integral part of the method. It is the selection of a suitable algorithm that may be crucial for specificity of measurements^21,22,40^. This is consistent with outcomes of a systematic review comprising 13 studies (277 patients) confirming that ACT tends to overstate sleep quality. The main limitation of the analysis is the absence of an algorithm for analyzing ACT results in critically ill patients, which is of crucial importance to measurement specificity^23^. Another factor affecting the results of monitoring is placement of the actigraph on the patient’s body. There are studies both stating that there is no significant difference between wrist and ankle ACT^41^ and showing that the opposite is true^42^.

Despite the numerous limitations, objective sleep assessment using ACT may be considered beneficial in ICUs even though experts have varied opinions on the role of this method. There is agreement, though, that such monitoring is supplemental and experimental and should not be used as a primary instrument for diagnosing sleep disorders^12,23,42^. Despite these pitfalls, ACT has been recognized as a method that may be used in the ICU setting to supplement other sleep quality assessment tools, for example when evaluating the effectiveness of sleep-promoting interventions or to use ACT for detecting delirium (study their physical activity patterns).

### Study limitations and recommendations

The main limitation of the study is its design (single-center nature, small patient sample). The ability to adequately cooperate on completing the RCSQ and the exclusion criteria limit the method’s reproducibility and applicability of results in the general population of critically ill patients (very frequent functional and structural CNS changes, nearly universal administration of sedatives, a high incidence of delirium, etc.). Here, objective sleep quality assessment is even more difficult. Another limitation is the absence of more detailed evaluation of physical activity during sleep. These results could provide interesting information in this patient group and should be challenging for future study. Another important limitation is the short actigraphy record. For valid and comprehensive sleep quality assessment, a multicenter randomized study is needed.

### Benefit to practice

- In ICUs, the absence of sleep of adequate quality and duration is a significant negative factor affecting the quality of care provided.
- Sleep quality assessment in ICU patients (both subjective and objective) is difficult and complicated and has a lot of limitations.
- The RCSQ is a relative simple, yet comprehensive and reliable instrument for subjective sleep quality assessment in the ICU setting. Although it has been widely used and validated in intensive care, factors limiting its universal applicability are not rare.
- The role of ACT as an instrument for objective sleep quality assessment in the intensive care setting is arguable. The main drawback of ACT is low reliability. However, it has potential as a supplemental method for sleep quality assessment in ICUs.

## CONCLUSION

In critically ill patients, sleep quality is very difficult to assess. The RCSQ appears to be a suitable and well-validated instrument for subjective sleep quality assessment. However, its use in the ICU setting is difficult and limited (as is the case with other questionnaires). On the other hand, ACT is a technically simple and available method, albeit with low reliability. Thus, the main potential of ACT is that it may serve as a supportive instrument to supplement other sleep quality assessment methods. For example, it may be used for evaluating the effectiveness of sleep-promoting interventions in ICUs.
